# Changes in contributions of different *Anopheles* vector species to malaria transmission in east and southern Africa from 2000 to 2022

**DOI:** 10.1186/s13071-023-06019-1

**Published:** 2023-11-07

**Authors:** Betwel J. Msugupakulya, Naomi H. Urio, Mohammed Jumanne, Halfan S. Ngowo, Prashanth Selvaraj, Fredros O. Okumu, Anne L. Wilson

**Affiliations:** 1 Environmental Health and Ecological Sciences Department, Ifakara Health Institute, PO Box 53, Ifakara, Tanzania; 2 Department of Vector Biology, Liverpool School of Tropical Medicine, Liverpool, UK; 3 School of Life Science and Bioengineering, The Nelson Mandela African Institution of Sciences & Technology, Arusha, Tanzania; 4 School of Biodiversity, One Health and Veterinary Medicine, University of Glasgow, Glasgow, UK; 5 Institute for Disease Modeling, Bill and Melinda Gates Foundation, Seattle, USA; 6 School of Public Health, Faculty of Health Sciences, University of the Witwatersrand, Park Town, Johannesburg, Republic of South Africa

**Keywords:** Malaria transmission, Entomological trends, *Anopheles*, East and southern Africa

## Abstract

**Background:**

Malaria transmission in Africa is facilitated by multiple species of *Anopheles* mosquitoes. These vectors have different behaviors and vectorial capacities and are affected differently by vector control interventions, such as insecticide-treated nets and indoor residual spraying. This review aimed to assess changes in the contribution of different vector species to malaria transmission in east and southern Africa over 20 years of widespread insecticide-based vector control.

**Methods:**

We searched PubMed, Global Health, and Web of Science online databases for articles published between January 2000 and April 2023 that provided species-specific sporozoite rates for different malaria vectors in east and southern Africa. We extracted data on study characteristics, biting rates, sporozoite infection proportions, and entomological inoculation rates (EIR). Using EIR data, the proportional contribution of each species to malaria transmission was estimated.

**Results:**

Studies conducted between 2000 and 2010 identified the *Anopheles gambiae* complex as the primary malaria vector, while studies conducted from 2011 to 2021 indicated the dominance of *Anopheles funestus*. From 2000 to 2010, in 57% of sites, *An. gambiae* demonstrated higher parasite infection prevalence than other *Anopheles* species. *Anopheles gambiae* also accounted for over 50% of EIR in 76% of the study sites. Conversely, from 2011 to 2021, *An. funestus* dominated with higher infection rates than other *Anopheles* in 58% of sites and a majority EIR contribution in 63% of sites. This trend coincided with a decline in overall EIR and the proportion of sporozoite-infected *An. gambiae*. The main vectors in the *An. gambiae* complex in the region were *Anopheles arabiensis* and *An. gambiae* sensu stricto (s.s.), while the important member of the *An. funestus* group was *An. funestus* s.s.

**Conclusion:**

The contribution of different vector species in malaria transmission has changed over the past 20 years. As the role of *An. gambiae* has declined, *An. funestus* now appears to be dominant in most settings in east and southern Africa. Other secondary vector species may play minor roles in specific localities. To improve malaria control in the region, vector control should be optimized to match these entomological trends, considering the different ecologies and behaviors of the dominant vector species.

**Graphical Abstract:**

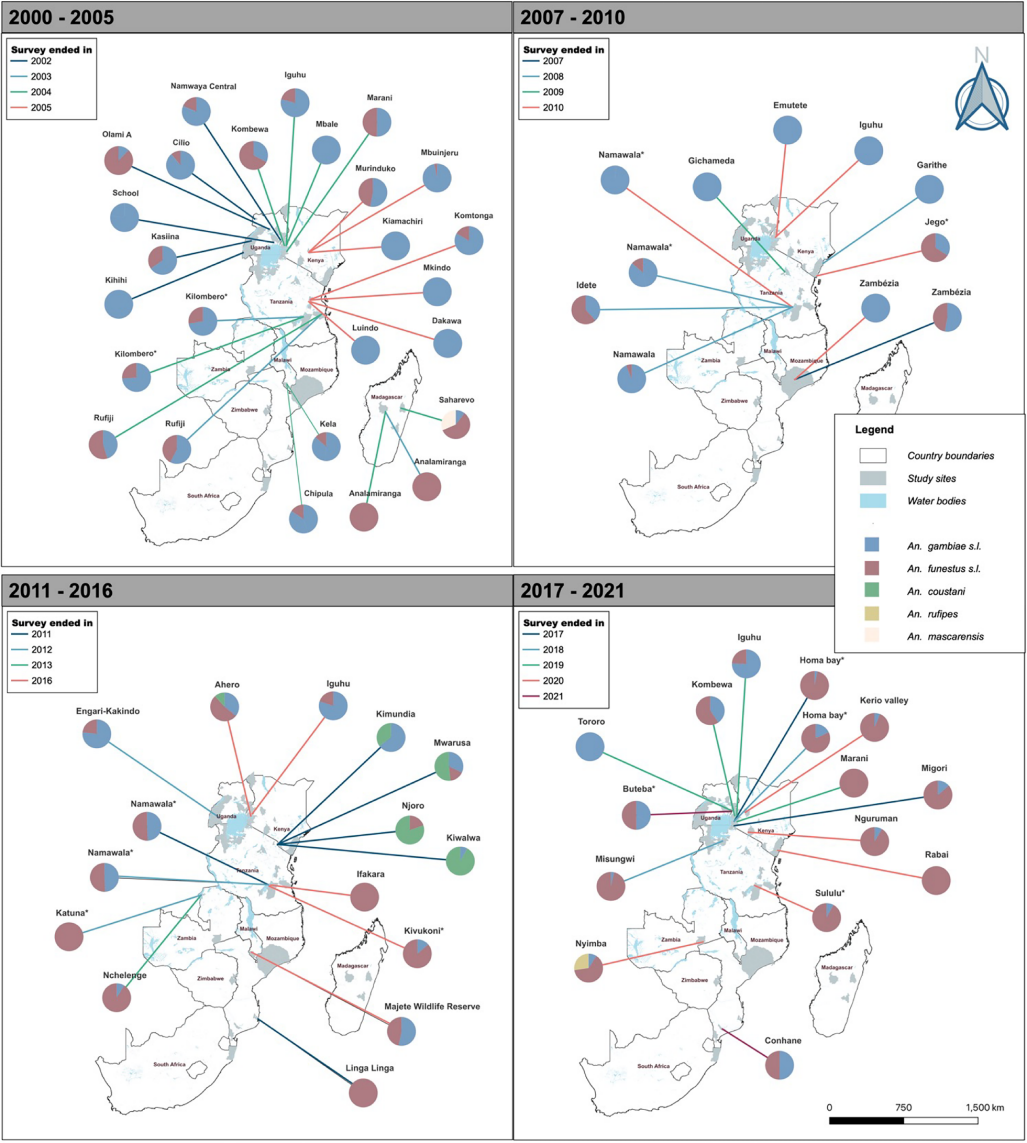

**Supplementary Information:**

The online version contains supplementary material available at 10.1186/s13071-023-06019-1.

## Background

Approximately 2 billion malaria cases and 12 million malaria deaths have been averted over the past two decades due to the scale-up of malaria interventions such as insecticide-treated nets (ITNs), indoor residual spraying (IRS), and effective case management [1]. ITNs, IRS, and case management were estimated to contribute 68%, 13%, and 19% of the decline in malaria cases, respectively, between 2000 and 2015 [2]. Unfortunately, malaria transmission persists, and in some settings there has been stagnation or even reversal of gains [3, 4]. The World Health Organization (WHO) estimates that, globally, we are 48% off the malaria control targets set in the Global Technical Strategy (GTS) (of 31 cases per 1000 population and 7.8 deaths per 1000 population by 2021) and that this situation could worsen [1, 5]. Current challenges include parasite mutations causing drug resistance and undetectability by rapid diagnostic tools [1, 6, 7], insecticide resistance in mosquitoes [8, 9], behavioral resilience or adaptation of the vectors [10–14], and human behaviors and occupational practices that expose people to infections [15, 16]. These challenges, coupled with the poor socioeconomic situation and weak health systems in endemic countries, mean that the ambitious targets set out in the GTS [5] will remain elusive without additional tools, efforts, and funding.

Malaria transmission in Africa is facilitated by different species of *Anopheles* mosquitoes, which have different behaviors and vectorial capacities. Generally, the four major vector species are *Anopheles gambiae*, *Anopheles funestus*, *Anopheles coluzzii*, and *Anopheles arabiensis*, which are the most anthropophilic *Anopheles* species in the world [10, 17]. In addition, several other species play an important but minor role in different localities, and in recent years, the Asian malaria vector *Anopheles stephensi* has also been spreading in Africa [18]. Because of their different behaviors around human dwellings, malaria vector species are affected differently by indoor insecticidal interventions, which currently dominate malaria control in Africa. For example, ITNs and IRS can effectively control populations of indoor-biting and indoor-resting species such as *An. gambiae* sensu stricto (s.s.) and *An. funestus* s.s. but are less effective against other species such as *An. arabiensis*, which readily bites non-human hosts and in outdoor settings [19, 20]. In fact, historical evidence from east and southern Africa suggests that *An. funestus* was likely the most important malaria vector prior to implementation of IRS as part of the Global Malaria Eradication Program which in some cases eliminated *An. funestus* from some areas and kept it at bay for several years [21–24]. More recent evidence suggests that with the wide-scale use of ITNs starting in the mid-2000s, the formerly dominant malaria vector, *An. gambiae* s.s., has been largely controlled in many parts of east and southern Africa [25–27]. Data from these areas also indicate a shift in both the composition and behavior of important malaria vector species [12, 23, 27–29], as well as increasing recognition of other vector species previously thought to be of secondary importance, such as *Anopheles parensis*, *Anopheles rivulorum,* and *Anopheles coustani* [28, 30–32].

These observations suggest the need to re-appraise the malaria transmission landscape and to better understand the dominant vector species in different settings across Africa. Understanding the characteristics of these vector species, their responsiveness to interventions, and their insecticide resistance profiles will be particularly important for any further progress in malaria control. This study aimed to conduct a systematic literature search and analyze the proportional contributions of different vector species to malaria transmission. Our focus was on the east and southern Africa regions, where indoor insecticidal interventions have historically been highly effective against major malaria vectors, notably *An. gambiae* and *An. funestus* [21–24]. The evidence review was limited to the period after 2000 when renewed malaria control efforts began following the formation of the Roll Back Malaria (RBM) Partnership in 1998 [33, 34] and the African leaders RBM summit in Abuja, Nigeria, in 2000 [35].

## Methods

### Literature search and compilation

A systematic search of published literature was conducted for articles describing malaria transmission by different vector species in Africa using three bibliographic databases, PubMed [36], Global Health [37], and Web of Science [38]. A combination of keywords and subject headings was used, including “sporozoite”, “sporozoite rate”, “entomological inoculation rate”, “EIR”, “*Anopheles*”, and “Africa” (Table 1). The search was limited to articles published between 1 January 2000 and 30 April 2023. The results were downloaded and imported into the EndNote reference manager [39], where duplicates were identified and removed.

**Table 1:** Search terms for literature review to determine the contribution of different *Anopheles* species to malaria transmission

Search ID	Search queries		
	PubMed	Global Health	Web of Science
S1	Sporozoite	Sporozoite	Sporozoite
S2	‘‘Sporozoites’’ [MeSH]	DE “Sporozoite”	Sporozoite rate
S3	Sporozoite rate	Sporozoite rate	Proportion with sporozoite
S4	Proportion with sporozoite	Proportion with sporozoite	Entomological inoculation rate
S5	Entomological inoculation rate	Entomological inoculation rate	EIR
S6	EIR	EIR	S1 OR S2 OR S3 OR S4 OR S5
S7	S1 OR S2 OR S3 OR S4 OR S5	S1 OR S2 OR S3 OR S4 OR S5 OR S6	*Anopheles*
S8	*Anopheles*	*Anopheles*	Africa
S9	‘‘*Anopheles*’’ [MeSH]	DE “*Anopheles*”	S6 AND S7 AND S8
S10	S7 OR S8	S8 OR S9	S6 AND S7 AND S8 (2000–2023)
S11	Africa	Africa	
S12	S6 AND S7 AND S8	S7 AND S10 AND S11	
S13	S6 AND S7 AND S8 (2000–2023)	S7 AND S10 AND S11 (2000–2023)	

Medical Subject Headings (MeSH) and Descriptor Index (DE) terms were used, where appropriate, to indicate subject headings in PubMed and Global Health, respectively. The Web of Science database does not allow the use of subject headings; thus only keywords were used to search for articles

### Inclusion and exclusion criteria

The articles were screened to identify those describing entomological inoculation rates (EIR) and the proportion of sporozoite-infected mosquitoes (sporozoite rate, SR) from entomological surveys conducted in the east and southern Africa regions. We included studies with data collected in either east Africa (Burundi, Kenya, Rwanda, Tanzania, and Uganda) or southern Africa (Botswana, Lesotho, Madagascar, Malawi, Mozambique, Namibia, South Africa, Eswatini, Zambia, and Zimbabwe) between January 2000 and April 2023. We included full-text articles or manuscripts reporting data from field surveillance of *Anopheles* vectors, including control or baseline data for intervention studies that separated such data from intervention data. Only studies with mosquito collection performed in both the rainy and dry seasons and those that reported the proportion of sporozoite-infected mosquitoes or EIR separately by species were considered. Studies were included if the primary vector group and complex (*An. funestus* sensu lato [s.l.] and *An. gambiae* s.l.) were both screened for sporozoites or if only one of them was tested because the other had either been collected in insignificant numbers or was not found. In addition, the studies had to have reported positive sporozoite infections for at least one of the *Anopheles* species tested.

Conversely, excluded studies consisted of mathematical modeling reports with no primary data, semi-field or laboratory studies, studies not conducted in east or southern Africa, those for which no surveillance dates had been given, and all studies conducted before 2000. Also excluded were studies reporting mosquitoes collected in only one season of the year, studies reporting only the overall proportion of sporozoite-infected mosquitoes and EIR instead of indicating the infections by vector species tested, studies reporting zero proportion of sporozoite-infected mosquitoes for all species tested, and studies that focused on one species despite multiple *Anopheles* species being collected in significant numbers. Studies where very few mosquitoes were tested for sporozoites relative to the number of mosquitoes collected (e.g., in one site, one mosquito was tested among 195 collected mosquitoes) and studies that combined intervention data and control data such that these could not be disaggregated into the proportion of control and treatment sporozoite-infected mosquitoes were also excluded.

### Data extraction

For each of the selected articles, the following data variables were extracted into a Microsoft Excel spreadsheet: study location (country, province, district, and village), latitude and longitude of the study site, the main vector control method(s) at the study site, dates of data collection, timing of rainy and dry seasons, number of collection nights, collection location (indoor/outdoor), collection method, method used to identify vectors (morphological, polymerase chain reaction [PCR]), proportion of sporozoite-infected mosquitoes, methods used to identify sporozoites (dissection, enzyme-linked immunosorbent assay [ELISA], or PCR), *Plasmodium* species, and EIR. Data on the proportions of female mosquitoes infected with any *Plasmodium* sporozoites were extracted to assess the infectivity of different malaria vectors. EIR data extracted were used to estimate the contribution of different vector species to malaria transmission.

Data on the proportion of sporozoite-infected mosquitoes and EIR data were extracted from selected articles to represent the smallest study unit presented in the articles (e.g., village or ward) for both rainy and dry seasons. For articles that had segregated data on sporozoite-infected mosquitoes or EIR indoors and outdoors, the estimates were aggregated and the parameters were estimated using the formulae below (see Eqs. 1 and 2). Where the sampling had been conducted for more than 1 year, the estimates for each year were extracted or estimated from the study data. Also, for studies that did not report EIR but provided components for its estimation, the estimation for each species was calculated as follows:

1 $$\mathrm {Sporozoite\, rate}= \mathrm  {\frac{Number \,of \,females \,with \, Plasmodium \,sporozoite \,infections}{Total \,number \,of \,female \,mosquitoes\,\, tested}}$$

2 $$\mathrm {Entomological\, inoculation\, rate\, (EIR)=Human \,biting \,\,rate \left(HBR\right)\times Sporozoite \,\,rate \left(SR\right)}.$$

All EIR estimates were annualized, considering the number of days or months during which data collection was performed. The recalculation of EIR was only done for studies that collected host-seeking mosquitoes. However, for those that collected resting mosquitoes, such as with pyrethrum spray catches (PSC), the EIR was extracted as presented in the article. On a few occasions, EIR data was not presented per species but overall EIR and percent contribution of each species to the EIR. In such instances, the percentage contribution was extracted as presented in the article, and EIR per species was calculated by multiplying the proportion of contribution by overall EIR.

### Data analysis

To estimate the contribution of different vectors to malaria transmission, the proportional contribution of species-specific EIR to the overall EIR in the study site was calculated using the formula:

3 $$ \mathrm  {Proportional\, contribution} =  \mathrm {\frac{EIR \,derived \,from \,a \,specific \,species \,of \,interest}{Total \,EIR \,of \,all \,species \,involved \,in \,malaria\, transmission \,at \,the \,study \,site}}$$

Mosquitoes were categorized into three groups: (i) *An. gambiae* s.l., corresponding to data presented for *An. gambiae* s.s., *An. arabiensis*, or *An. merus*, and when members of *An. gambiae* s.l. were unspecified; (ii) *An. funestus* s.l. corresponding to data presented for *An. funestus* s.s. and when members of *An. funestus* s.l. were unspecified; and (iii) other secondary vectors corresponding to other *Anopheles* species. Both EIR and *Plasmodium* sporozoite infection data were tabulated by study date and sites. The *ggplot2* package [40], implemented in R statistical software [41], was used to plot the proportions of sporozoite-infected mosquitoes over time, using scatter plots. Smooth trend lines were added using the generalized additive method. Using QGIS (Quantum Geographical Information System) software [42], maps were created to illustrate the proportional contribution of different vector species in the different study sites in east and southern Africa for the periods 2000–2010 and 2011–2021.

## Results

**Fig. 1:**
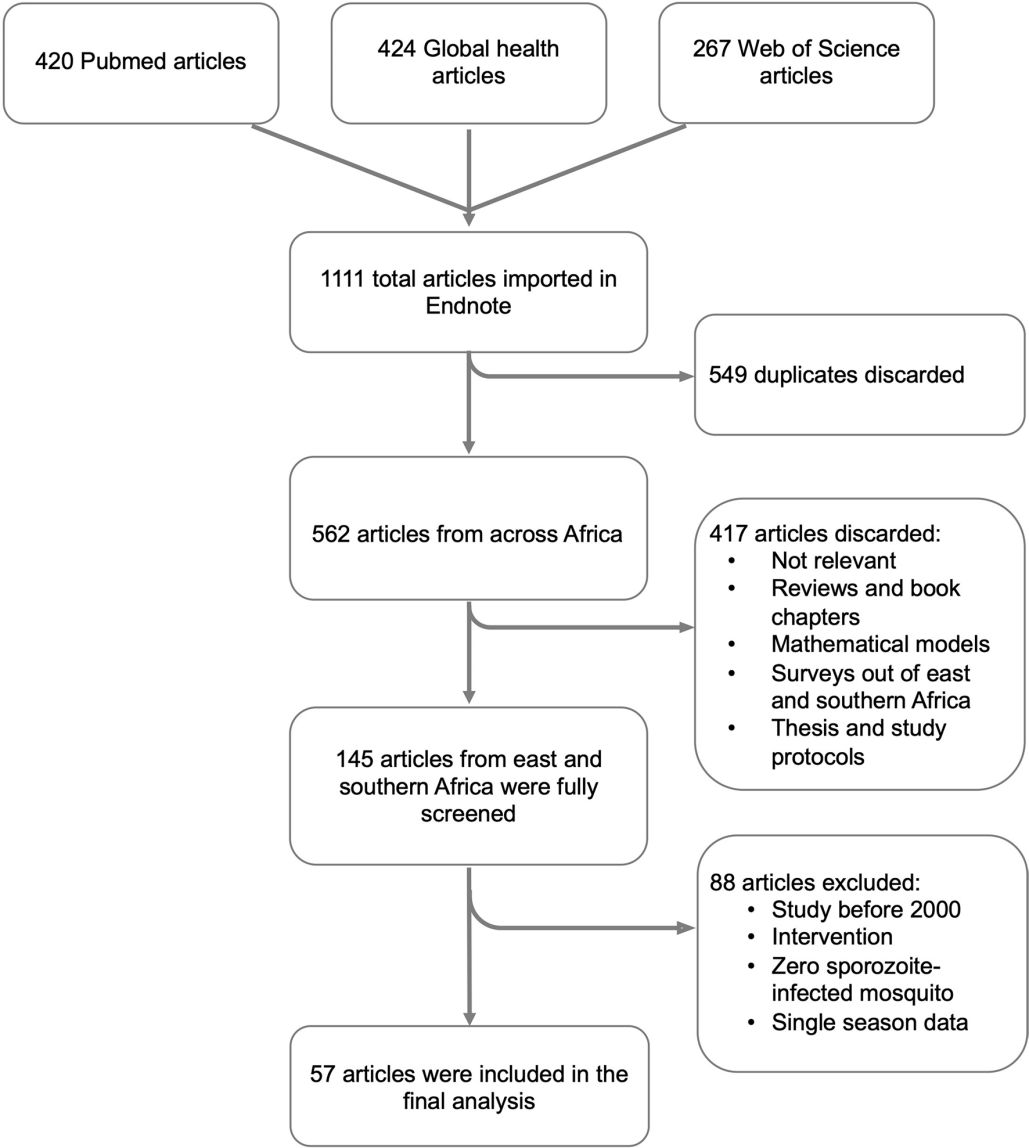
Flow chart of the article search and screening process

A total of 1111 articles were obtained from the literature search, of which 549 duplicates were screened out. An additional 417 articles were removed because the studies did not meet the inclusion criteria. The remaining 145 articles were subjected to full-text scrutiny, and 57 articles were included in the final analysis (Fig. 1).

### Study characteristics

The studies included in this analysis were conducted in nine African countries: Kenya (*n* = 23), Madagascar (*n* = 3), Malawi (*n* = 2), Mozambique (*n* = 5), South Africa (*n* = 1), Tanzania (*n* = 13), Uganda (*n* = 5), Zambia (*n* = 4), and Zimbabwe (*n* = 1) (Table 2). Data presented in the studies were collected between 2000 and 2021, and contained a total of 113 unique data points representing different sites and times of data collection. Extraction of sporozoite data resulted in 105 data points, and extraction of EIR created 67 data points. Mosquitoes were collected using different trapping methods: in the majority of studies (*n* = 32, containing 63 data points), collection was only performed indoors (Table 2). Twenty-four studies collected mosquitoes both indoors and outdoors (containing 46 data points) and two studies collected mosquitoes only outdoors (containing four data points). Most studies used either Centers for Disease Control and Prevention (CDC) light traps or PSC (*n* = 51, containing 97 data points). These traps were used alone or together, or supplemented with other trapping methods to collect both indoor biting and resting mosquitoes. CDC light traps were also common for the collection of outdoor biting mosquitoes (used in 13 of 30 studies that collected mosquitoes outdoors). The other methods used included mechanical and mouth aspirators (*n* = 10), pit shelters (*n* = 9), human landing catches (HLC, *n* = 8), clay pots (*n* = 5), exit traps (*n* = 3), BG-Suna traps, (*n* = 2) Furvela tent traps (n = 1), and artificial resting boxes (*n* = 1). Between 2000 and 2010, indoor collection was typically performed using PSC (27 data points, 51.9%) or CDC light traps (20 data points, 38.5%). Between 2011 and 2022 there was greater use of CDC light traps (37 data points, 94.9%) but PSC still played a role (29 data points, 50.9%). ELISA was the most common method used to test for sporozoite infections in mosquitoes (*n* = 45 studies, containing 87 data points). PCR was used in 11 studies (containing 21 data points), and only two studies (containing five data points) used dissection to detect sporozoites (Table 2). The methods used to identify mosquitoes differed between the periods 2000–2010 and 2011–2022. There was an increase in the use of molecular methods for mosquito identification, from 75% (*n* = 39) of the data points for *An. gambiae* s.l. and 15% (*n* = 8) for *An. funestus* in 2000–2011 to 92% (*n* = 56) of the data points for *An. gambiae* s.l. and 74% (*n* = 45) for *An. funestus* in 2011–2021 (Additional file 1: Table S1). Only 48% of the data points in the 2000–2010 period were identified in the articles as having vector control interventions in place, which included mainly ITNs, IRS, and untreated bed nets; in the remaining data points in that period, either studies reported having no intervention or publications did not provide data on vector control interventions in place. From 2011 to 2022, all data points reported vector control interventions in the study sites, which included ITNs and IRS, and in one study larvicidal and untreated bed nets (Additional file 1: Table S1).

**Table 2:** Characteristics of studies included in the analysis

Country	Position	Trap used indoors	Trap used outdoors	Sporozoite detection	Data points	Citation
Kenya	Indoor	CDC		ELISA	1	[43]
		CDC and PSC		ELISA	3	[44]
				PCR	1	[45]
		PSC		ELISA	17	[46–53]
				PCR	8	[54–56]
	Indoor and outdoor	CDC and PSC	CDC	ELISA	1	[57]
			CDC and pit shelter	ELISA	2	[58]
			CDC, HLC, clay pot, and pit shelter	PCR	1	[45]
		CDC and Prokopack	CDC	ELISA	5	[59]
		CDC and mouth aspirators	CDC	ELISA	4	[28]
		CDC, HLC and PSC	CDC, HLC, clay pots, and pit shelter	PCR	1	[60]
		PSC	Clay pots	ELISA	1	[61]
		PSC and Prokopack	Prokopack, clay pots, and pit shelter	ELISA	2	[62]
		PSC and rotator traps	Rotator traps	ELISA	6	[63]
	Outdoor		CDC (CO2 Baited)	PCR	3	[64]
Madagascar	Indoor and outdoor	CDC (CO2 baited)	CDC (CO2 Baited)	ELISA	1	[65]
		HLC and PSC	HLC and pit shelter	ELISA	3	[66, 67]
Malawi	Indoor	PSC		PCR	2	[68]
	Indoor and outdoor	BG-Suna traps	BG-Suna traps	PCR	1	[69]
Mozambique	Indoor	CDC and exit traps		ELISA	1	[70]
		Resting collection		Dissection	1	[71]
		Window exit traps		PCR	2	[72]
	Indoor and outdoor	CDC, mouth aspirators, and exit traps	Furvela tent traps	ELISA	1	[73]
		HLC	HLC	ELISA	1	[74]
South Africa	Outdoor		Clay pots	ELISA	1	[31]
Tanzania	Indoor	CDC		Dissection	4	[75]
				ELISA	13	[26, 27, 76–79]
		CDC and PSC	Pit shelter	ELISA	3	[80]
		CDC and Prokopack		ELISA	1	[81]
		CDC and backpack aspirators		ELISA	1	[82]
	Indoor and outdoor	CDC	CDC and Prokopack	ELISA	2	[83]
		CDC and HLC	BG-Suna traps and HLC	ELISA	1	[84]
		CDC, Mouth aspirators, Backpack aspirators	Backpack aspirators, artificial resting boxes	ELISA	1	[85]
Uganda	Indoor	CDC		ELISA	1	[86]
		Prokopack		PCR	1	[87]
	Indoor and outdoor	CDC, HLC, Prokopack	HLC and pit shelter	ELISA	1	[88]
		HLC	HLC	ELISA	6	[89]
		HLC, PSC, mouth aspiration	HLC	ELISA	1	[90]
Zambia	Indoor	CDC		ELISA	1	[91]
				PCR	1	[91]
		CDC and PSC		ELISA	1	[92]
	Indoor and outdoor	CDC	CDC	ELISA	1	[93]
		CDC and PSC	CDC	ELISA	1	[94]
Zimbabwe			CDC and pit shelter	ELISA	2	[95]

### Proportion of mosquitoes infected with *Plasmodium* sporozoites

Of the 105 data points that contained data on the proportion of *Plasmodium*-infected mosquitoes, 46 were from studies conducted between 2000 and 2010, and 59 were from 2011 to 2021. Of the 113 data points, only 89 reported the species of *Plasmodium* identified in mosquitoes. The most common *Plasmodium* species was *Plasmodium falciparum*, which was found alone in 83 data points, and in a few studies it was reported to be present with other *Plasmodium* species such as *P. malariae*, *P. vivax*, or *P. ovale* (six data points). There was no trend in *Plasmodium* species over time.

In studies that collected data between 2000 and 2010, members of *An. gambiae* s.l. had the highest proportions of sporozoite infections in 56.5% (*n* = 26) of the sites while only in 43.5% (*n* = 20) of the sites *An. funestus* s.l. had the highest infection proportions. The proportion of infected *An. gambiae* s.l. ranged between zero and 17.4% (median = 1.4%), while other vectors including members of *An. funestus* s.l. ranged between zero and 6.3% (median = 1.5%). On the other hand, in studies conducted between 2011 and 2021, members of *An. funestus* had the highest proportion of sporozoite infections in 57.6% (*n* = 34) of the sites, while *An. gambiae* s.l. had the highest proportion of sporozoite infections in only 28.8% (*n* = 17) of the sites. In this period, there was one (1.7%) site where *An. gambiae* and *An. funestus* had equal proportions of sporozoite-infected mosquitoes and seven (11.9%) other sites where vector species other than *An. gambiae* or *An. funestus* had the highest proportion of sporozoite infections. In studies conducted between 2011 and 2021, the proportion of infected mosquitoes ranged between zero and 26.4% (median = 2.0%) among all members of *An. funestus* s.l., 0 and 15% (median = 0.8%) among *An. gambiae* s.l., and between 0 and 9.1% (median = 0.4%) among the secondary vectors (Table 3, Additional file 1: Table S1).

**Table 3:** Summary of number of sites and species involved in the assessment of sporozoites and number of sites where these species had the highest proportions of sporozoite-infected mosquitoes.

Period	Species	Number of sites where species were tested	Range of proportion of sporozoite-infected mosquitoes (%)	Median (%)	Number of sites with highest sporozoite proportions for each tested species
2000–2010	*Anopheles gambiae* s.l.	23	0.02–7.7	1.5	11
	*Anopheles gambiae* s.s.	15	0–15.3	2.8	9
	*Anopheles merus*	4	0–17.4	2.2	2
	*Anopheles arabiensis*	22	0–11.1	0.3	4
	All *Anopheles gambiae* complex	64	0–17.4	1.4	26
	*Anopheles funestus* s.l.	37	0–6.3	1.1	17
	*Anopheles funestus* s.s.	5	0–5.2	3.0	3
	All *Anopheles funestus* complex	42	0–6.3	1.5	20
	*Anopheles coustani*	2	0	−	0
	*Anopheles rufipes*	1	0	−	0
	*Anopheles mascarensis*	3	0–0.7	0	0
	Other *Anopheles*	3	0	−	0
	Sites without sporozoite data				6
	Subtotal of data points				52
2011–2021	*Anopheles gambiae* s.l.	32	0–15	1.0	12
	*Anopheles gambiae* s.s.	16	0–13.4	2.2	5
	*Anopheles arabiensis*	22	0–8.3	0.3	0
	All *Anopheles gambiae* complex	70	0–15	0.8	17
	*Anopheles funestus* s.l.	35	0–13.9	0.8	18
	*Anopheles funestus* s.s.	22	0–26.4	3.2	15
	All *Anopheles funestus* complex	57	0–26.4	2.0	33
	*Anopheles parensis*	6	0–1.4	0.8	1
	*Anopheles coustani*	16	0–7	1.1	6
	*Anopheles rufipes*	3	0–9.1	2.9	1
	Other *Anopheles*	6	0–7.6	0.1	0
	Sites where members of *Anopheles funestus* s.l. and *Anopheles gambiae* s.l. had equal proportions of sporozoite-infected mosquitoes				1
	Sites without sporozoite data				2
	Subtotal of data points				61

All *An. gambiae* complex and all *An. funestus* complex show the summary of sporozoite-infected mosquitoes of all members of each complex or group from each data point irrespective of whether they were identified to species

**Fig. 2:**
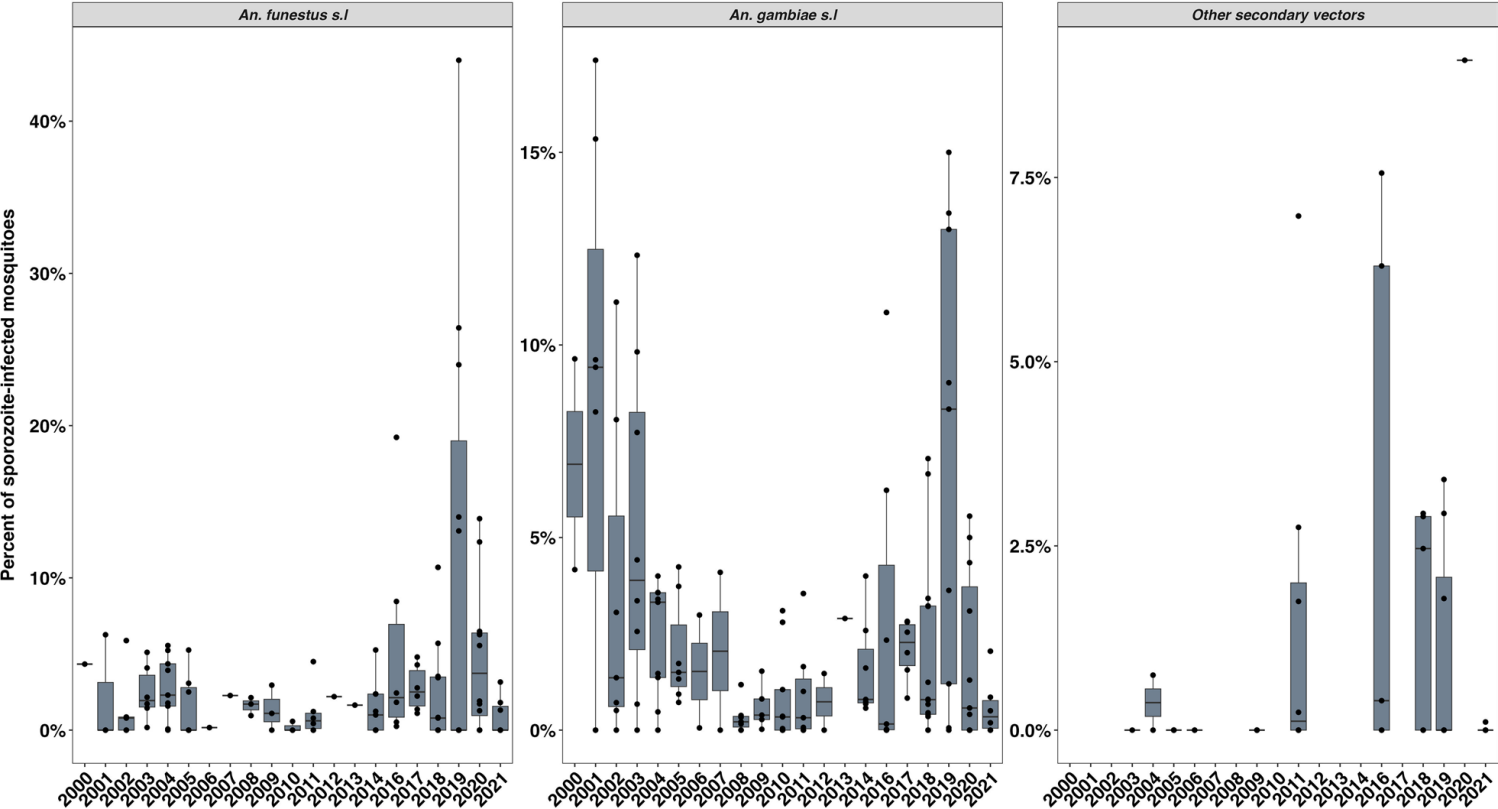
The proportion of sporozoite-infected mosquitoes in the study sites from 2000 to 2021

We detected an overall drop in the proportion of sporozoite-infected mosquitoes among *An. gambiae* s.l. but no discernible decline in *An. funestus* between 2000 and 2021. This is, however, without considering the proportion of sporozoite-infected *An. funestus* and *An. gambiae* in 2019, which were exceptionally high and most were from a single study. Due to fewer data points presented for secondary vectors from the published articles, no clear trend could be observed (Fig. 2).

In studies where the members of *An. gambiae* complex and *An. funestus* group were molecularly distinguished and their sporozoite infections reported, the most common sibling species of *An. gambiae* complex were *An. arabiensis* and *An. gambiae* s.s., and on fewer occasions *An. merus*, while the most common members of the *An. funestus* group were *An. funestus* s.s., and on fewer occasions *An. rivulorum*, *An. leesoni*, and *An. longipalpis*.

### The relative contribution of different vector species to malaria transmission

**Fig. 3:**
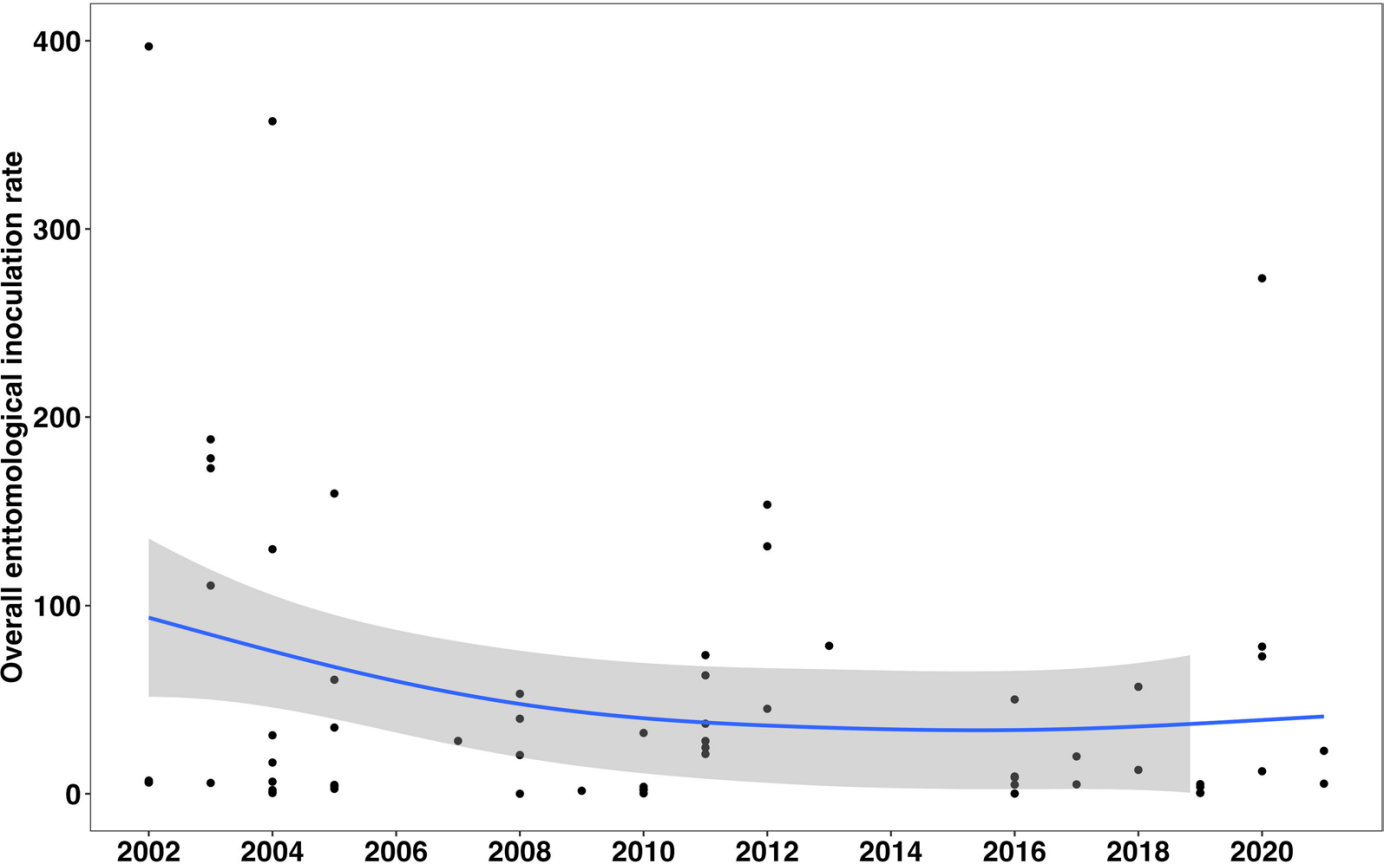
The trend in the overall entomological inoculation rate for different data points collected between 2000 and 2021 in the included studies

**Fig. 4:**
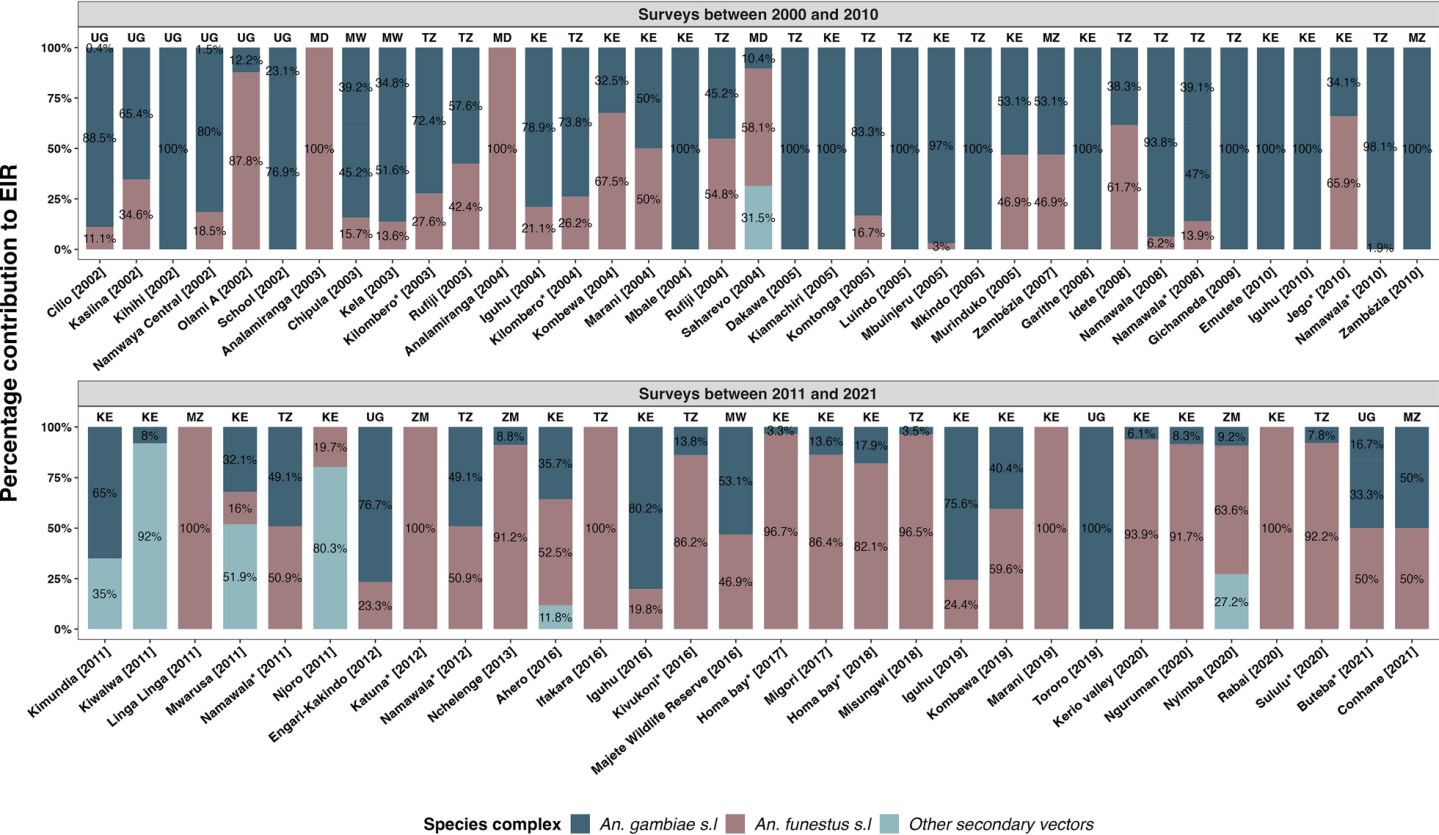
Relative contributions of malaria vectors from different east and southern Africa sites based on studies conducted between 2000 and 2021. EIR denotes entomological inoculation rate, which is the number of infectious bites an individual receives per unit of time. The * sign on the names in the *x*-axis indicates that more than the mentioned study sites were involved in the survey, while the final year of the survey is indicated in brackets. On top of the bars are abbreviations of countries: *KE* Kenya, *MD* Madagascar, *MW* Malawi, *MZ* Mozambique, *SA* South Africa, *TZ* Tanzania, *UG* Uganda, *ZM* Zambia, *ZW* Zimbabwe

**Fig. 5:**
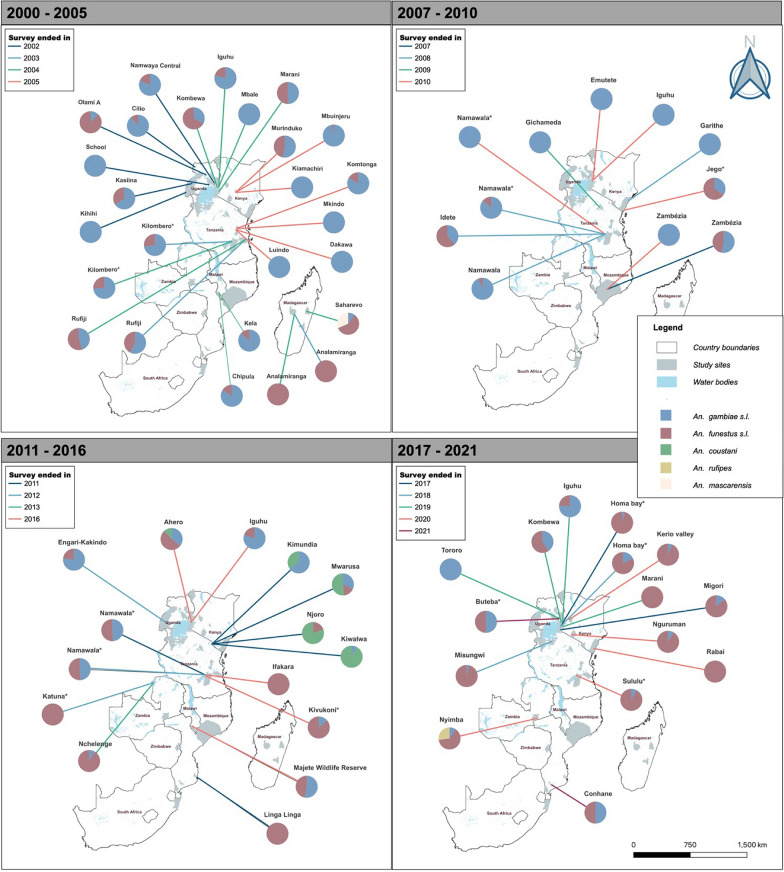
Locations of the study sites, along with the respective contributions of different vectors to malaria transmission (a) between 2000 and 2005, (b) between 2006 and 2011, (c) between 2012 and 2017, and (d) between 2018 and 2021. The years marked on the map signify the final year of data collection as reported in the studies included

Analysis suggests a decline in overall EIR (all *Anopheles* combined) in recent years relative to the early 2000s (Fig. 3). This decline has been experienced with changes in the contribution of different species in malaria transmission. Multiple *Anopheles* vectors have contributed to malaria transmission, with a major shift occurring between 2010 and 2012 when the dominance of *An. gambiae* began fading (Fig. 4). In the period from 2000 to 2010, most studies reported that the EIR contribution was primarily from members of *An. gambiae* s.l.. In 28 out of 37 sites, members of *An. gambiae* s.l. contributed more than 50% to the overall EIR (Fig. 4). Conversely, only 8 of the 37 sites had other *An. funestus* as the majority contributors to the overall EIR, and one site with an equal contribution between *An. gambiae* s.l. and *An. funestus* s.l. Since 2011, however, there has been a decrease in the contribution of *An. gambiae* s.l. to the overall EIR. In 19 out of 30 studies, *An. funestus* mosquitoes contributed more than 50% to the EIR. Conversely, only six and another three of the 30 sites had members of *An. gambiae* s.l. and other secondary vectors contribute more than 50% to the EIR, respectively. Two sites had equal contributions between *An. gambiae* s.l. and *An. funestus* s.l. Furthermore, members of *An. funestus* were more important than other secondary vectors in various sites in east and southern Africa (Fig. 5).

## Discussion

The main Afro-tropical malaria vectors include *An. gambiae*, *An. arabiensis*, *An. funestus*, and *An. coluzzii*, which all play a major role in malaria transmission across Africa. In the past decade, the widespread use of indoor insecticidal interventions, notably ITNs and IRS, may have impacted the vector species differently due to their different behaviors, and possibly led to changes in the dominance between these vectors in malaria transmission [27, 82]. This analysis was conducted to systematically compile reports of entomological surveys conducted between 2000 and 2022 to assess the roles of different vectors in malaria transmission in east and southern African countries. The main finding was that the contribution of *An. funestus* to malaria transmission has become more pronounced than in previous decades, while the role of the formerly dominant malaria vector, *An. gambiae*, appears to have declined. The increasing importance of *An. funestus* may not be a new phenomenon, as *An. funestus *may have been the most important vector before the Global Malaria Eradication Program. Currently, *An. funestus* is increasingly becoming the major contributor to malaria transmission across multiple sites within the region, as its proportion of sporozoite-infected mosquitoes and proportional contribution to EIR now consistently exceeds those of *An. gambiae* s.l. We also observed a decrease in the EIR and the proportion of sporozoite-infected *An. gambiae* but no obvious decrease in the proportion of sporozoite-infected *An. funestus* between 2000 and 2021.

We postulate that increased coverage of insecticidal indoor vector control interventions and the differential susceptibility of *An. gambiae* and *An. funestus* to these interventions may have led to the increasing contribution of *An. funestus* to malaria transmission observed in this study. Increased funding in the late 2000s and early 2010s [1, 96] led to the rollout of insecticidal vector control interventions, predominantly ITNs and to a lesser extent IRS, across sub-Saharan Africa. Similar patterns are seen in the included studies with reported use of insecticidal vector control interventions in less than half the data points included pre-2011, increasing to all studies between 2011 and 2022. Studies across Africa indicate that ITNs are effective against mosquitoes such as *An. gambiae* s.s. that mostly prefer to bite humans inside houses [25–27]. *Anopheles funestus* largely shares these behaviors, and therefore it should be expected that the scale-up of ITNs across Africa from the early twenty-first century should have effectively controlled both *An. funestus* and *An. gambiae*. However, indicators in this study show that the importance of *An. funestus* in malaria transmission has now become more noticeable even in areas where *An. funestus* is outnumbered by other vectors [81, 82]. *Anopheles funestus* is strongly resistant to pyrethroid insecticides used in ITNs, and in several settings insecticide resistance developed earlier and more rapidly in *An. funestus* than other vector species [97–101], perhaps explaining why they may have been less impacted by ITNs. Also, several other traits give *An. funestus* an advantage in malaria transmission by increasing the risk of the vector contracting *Plasmodium* parasites. Among these are its high anthropophilic tendency, the ability to survive longer [17, 102, 103], and a greater tendency to take multiple blood meals to complete a single gonotrophic circle (Jumanne, unpublished). In addition, *An. funestus* tends to rest in areas that are out of reach of indoor interventions [104], and tends to seek blood meals in the early morning or evening when humans are unprotected [11, 14]. Changes in entomological procedures such as ELISA during the last few years may have also contributed to the reduction in EIR observed over time. In the 2010s, changes were made to ELISA procedures, where boiling of the lysate at 100 °C for 10 min was recommended [105]. This was to reduce false positives, since the ELISA method had previously been sensitive to protozoans, including non-*Plasmodium* parasites [105].

In addition to the primary vectors, secondary vectors play a part in malaria transmission across east and southern Africa. In this review, we aimed to assess the relative importance of vector species across the region and thus only included studies that assessed sporozoite infections in multiple species when more than one species was collected in one site. This meant that we excluded several studies indicating the involvement of different secondary vectors such as *An. vaneedeni* and *An. rivulorum* in malaria transmission [31, 32, 106], but which were silent about the importance of other collected vectors. Several studies indicated the importance of secondary vectors such as *An. coustani* in specific locations, including some studies reporting an unexpectedly high contribution of secondary vectors to malaria transmission, mainly contributed by mosquitoes collected outdoors [28]. This should thus be treated with caution due to the inconsistent and unexpected nature of the contribution of the secondary vector. The majority of studies did not test secondary vectors for sporozoites, so it was difficult to gauge the trend of secondary vectors in malaria transmission in this review. More recently, *An. stephensi*, an invasive urban malaria vector, has been identified in East Africa with the potential to increase malaria transmission [18, 107]. It will be important to expand surveillance for this species and determine its relative contribution to malaria transmission alongside native vector species.

We observed large differences in how data were reported across studies, which made it challenging to pool published data to obtain averages over time and space. We therefore call on researchers to report results in a way that discloses details of spatial and temporal variability in vectors to be able to pinpoint where and which species is important. This includes (i) indicating dates of the survey; (ii) proper description of the study sites (georeferences, ecology and economic activities, the timing of the seasonal rains, interventions used and coverage, and dates of intervention campaigns); (iii) proper mosquito identification (morphological and molecular identification to confirm and identify sibling species); (iv) full report of how different vector species were treated in the survey; (v) if more than one site (village) was involved, separating the results for each site to enable other researchers to identify the spatial variability in the estimates; and (vi) reporting mosquitoes collected by different traps separately.

This study had several limitations. Firstly, there were several sites in the east and southern Africa region where malaria is endemic but there was either a very small number of studies or no studies at all with entomological data on malaria transmission. Most of the studies included in this review were conducted in Kenya and Tanzania, implying that while this systematic review may be strongly indicative of the trends, it does not fully represent the overall picture of the role of different vectors in the region. Secondly, in most of the studies, the EIR or sporozoite-infected proportions of mosquitoes were estimated from only *An. gambiae* s.l. and *An. funestus* s.l. Thus, it is likely that the importance of other secondary vectors remains less well understood and may have been underrepresented. Third, the studies considered involved the use of a diverse set of methods for trapping, trapping locations (indoors, outdoors, or both), and detection of sporozoites (ELISA, PCR, dissection). Several studies tested individual mosquitoes for sporozoites while others used subsamples or tested mosquitoes in pools. All these methods have different sensitivity and may introduce biases in estimating the importance of vector species. However, it was difficult to segregate the reviewed articles by method; thus, the analysis was conducted for all articles. Fourth, in the studies included in the review, we noticed a move away from morphological identification and a rise in the use of molecular approaches for mosquito identification over time. This may contribute to bias, since morphological identification may have misclassified vectors. We were unable to extract data for specific species within complexes or groups due to discrepancies in identification procedures. As a result, the results of this study are mostly represented as *An. gambiae* s.l. (for which the most dominant members were *An. gambiae* s.s. and *An. arabiensis*) and *An. funestus* group (for which the dominant is *An. funestus* s.s.). Lastly, this review did not assess how the importance of different vectors may vary across different ecological conditions. There may have been ecological changes over time which may for example have increased habitat suitability for *An. funestus* or decreased habitat suitability for *An. gambiae* s.l.

Given the apparent rising importance of *An. funestus* in east and southern Africa, new vector control interventions will be required in addition to ITNs and IRS. This may include sterile insect techniques [108], genetic modification of mosquitoes [109], attractive targeted sugar baits [110–112], space spraying of mosquito swarms [113, 114], and spatial repellents [115, 116]. However, in the meantime, as the majority of these interventions are still under development, the available methods should be deployed innovatively and judiciously, including IRS with effective insecticides such as organophosphate and neonicotinoids (to which most vectors including *An. funestus* remain susceptible [18, 97, 101]), new ITNs with dual active ingredients, expanded use of larval source management (LSM), or combining ITNs or IRS with LSM.

## Conclusions

In this review, we compiled reports of entomological surveys assessing malaria transmission. The proportional contribution of different vector species has changed significantly over the past 20 years. As the role of *An. gambiae* has declined, *An. funestus* now appears to be dominating most settings in east and southern Africa. Other secondary vector species may be playing minor roles in specific localities. To achieve greater improvements in malaria control in these areas, vector control should be optimized to match these entomological trends, taking into account the different ecology and behaviors of the dominant vector species. While innovative methods are being developed, currently available tools should be enhanced, including next-generation ITNs and IRS, and LSM.

### Supplementary Information

**Additional file 1**. The contribution of different vectors in malaria transmission for each datapoint, along with data collection dates, ecology, interventions, and identification methods used.

## Data Availability

The datasets used and/or analyzed during the current study are available from the corresponding author upon reasonable request.

## Abbreviations

CDC: Centers for Disease Control and Prevention
EIR: Entomological inoculation rate
ELISA: Enzyme-linked immunosorbent assay
GTS: Global Technical Strategy
HLC: Human landing catches
IRS: Indoor residual spraying
ITNs: Insecticide-treated nets
LSM: Larval source management
PCR: Polymerase chain reaction
PSC: Pyrethrum spray catch
QGIS: Quantum geographical information system
RBM: Roll Back Malaria
WHO: World Health Organization
